# The population genomics of begomoviruses: global scale population structure and gene flow

**DOI:** 10.1186/1743-422X-7-220

**Published:** 2010-09-10

**Authors:** HC Prasanna, D P Sinha, Ajay Verma, Major Singh, Bijendra Singh, Mathura Rai, Darren P Martin

**Affiliations:** 1 Indian Institute of Vegetable Research, P B No. 1, P O - Jakhini, Shahanshapur, Varanasi, India; 2 Dorectorate of Wheat Research, P B NO. 158, Aggrasain Marg, Karnal, India; 3 Institute of Infectious Diseases and Molecular Medicine, University of Cape Town, South Africa; 4 Department of Plant sciences, Mail Stop 3, One Shields Avenue, University of California, Davis, 95616, California, USA

## Abstract

**Background:**

The rapidly growing availability of diverse full genome sequences from across the world is increasing the feasibility of studying the large-scale population processes that underly observable pattern of virus diversity. In particular, characterizing the genetic structure of virus populations could potentially reveal much about how factors such as geographical distributions, host ranges and gene flow between populations combine to produce the discontinuous patterns of genetic diversity that we perceive as distinct virus species. Among the richest and most diverse full genome datasets that are available is that for the dicotyledonous plant infecting genus, *Begomovirus*, in the Family Geminiviridae. The begomoviruses all share the same whitefly vector, are highly recombinogenic and are distributed throughout tropical and subtropical regions where they seriously threaten the food security of the world's poorest people.

**Results:**

We focus here on using a model-based population genetic approach to identify the genetically distinct sub-populations within the global begomovirus meta-population. We demonstrate the existence of at least seven major sub-populations that can further be sub-divided into as many as thirty four significantly differentiated and genetically cohesive minor sub-populations. Using the population structure framework revealed in the present study, we further explored the extent of gene flow and recombination between genetic populations.

**Conclusions:**

Although geographical barriers are apparently the most significant underlying cause of the seven major population sub-divisions, within the framework of these sub-divisions, we explore patterns of gene flow to reveal that both host range differences and genetic barriers to recombination have probably been major contributors to the minor population sub-divisions that we have identified. We believe that the global *Begomovirus *population structure revealed here could facilitate population genetics studies into how central parameters of population genetics namely selection, recombination, mutation, gene flow, and genetic drift shape the global begomovirus diversity.

## Background

The study of genome-wide patterns of sequence variation within and between closely related virus species can be used to efficiently infer the fine-scale genetic structures of virus populations. Information on population structures - particularly that pertaining to stratification and admixture (i.e. gene flow) - is valuable in a variety of situations. These include the establishment of sensible species/subspecies/strain classification criteria, the detection of geographical or biological barriers to gene flow, and the identification of demographic, epidemiological or evolutionary processes responsible for virus differentiation [1-3]. More specifically, a detailed knowledge of virus population stratification can provide important insights into how virus genetic diversity generated through mutation and recombination is shaped into discernable taxonomic groupings: A process that involves natural selection and genetic drift in the context of epidemiological fluctuations in virus population sizes and the spatial movement of viruses across land-masses [4,5]. The deeper understanding of virus epidemiology and evolutionary history that can potentially be provided by studies of virus population structure is also directly applicable to the formulation of strategies for controlling the dissemination of viral diseases [6,7].

It is therefore surprising that there have been no studies specifically aimed at identifying global-scale population genetic structures within agriculturally significant groups of plant pathogenic viruses such as the geminiviruses, potyviruses, tospoviruses, cucumoviruses and sobemoviruses. For example, virtually nothing is known about population stratification amongst the various geminivirus species within the genus *Begomovirus *that are responsible for economically devastating diseases of many leguminaceous, solanaceous, curcurbitaceous and malvaceous crop species throughout the tropical and subtropical regions of the world [8-15]. Begomoviruses are transmitted by the whitefly, *Bemasia tabaci*, and have circular single stranded one (i.e. monopartite) or two (i.e. bipartite) component genomes ranging in size from ~2.7 Kb (for monopartite species) to ~5.4 Kb (for bipartite species) [16].

Relationships amongst DNA-A and DNA-A-like sequences are widely used in formalized begomovirus species, strain and variant demarcation schemes [17-19]. Based on the phylogenies of currently sampled DNA-A and DNA-A-like sequences, begomoviruses have been classified worldwide into seven different groups. Whereas begomoviruses originating from the Old World have been divided into Africa-Mediterranean, Indian, Asian, and legume-infecting viruses (legumoviruses), those originating in the New World have been classified into Latin American and Meso American groups. A seventh group of Sweet potato-infecting viruses (swepoviruses) is found in both the Old and New Worlds [20]. This phylogenetic sub-division of the begomoviruses broadly corresponds with their geographical distributions [20] except that the divergent legumovirus and swepovirus [20,21] lineages occur alongside other distantly related begomovirus groups.

The current *Begomovirus *taxonomic classification system is based almost entirely on traditional phylogenetic reconstruction and pairwise genetic distance estimators (such as Hamming or p-distances) [17-20,22]. These estimators have been commonly used because of both their simplicity and their relatively unambiguous approximation of relationships between sequences.

However, frequent inter-species genetic recombination is a prominent feature of begomovirus evolution [22-27] that can obscure estimated relationships amongst groups of species [28-30] and can thus undermine the robustness of current classification schemes. In this regard it is noteworthy that population genetic analysis based approaches can in many cases explicitly account for genetic recombination. In fact, enumerating the exchange of genetic material between individuals is the foundational basis of some population genetic methods that seek to describe the degrees to which different partially isolated sub-populations within structured meta-populations interact with one another.

Here we use such a population-genetics model-based clustering approach both to verify the existence of defined sub-populations within the global begomovirus meta-population and to track the movement of genetic material between these populations. Besides identifying hitherto unappreciated genetically discreet begomovirus sub-populations, our study provides interesting insights into how constraints on genetic recombination imposed by geographical distance and/or host range differences may contribute to taxonomically relevant patterns of begomovirus diversity.

## Results

### Assessment of linkage disequilibrium

The admixture model implemented in STRUCTURE assigns individual genomes to populations under the assumption that all polymorphic sites within the genomes are in linkage equilibrium. We therefore tested the degree of linkage equilibrium that is evident within begomovirus genomes using LIAN 3.4 to calculate a standardized index of association between genome sites (I^S^_A_). Monte Carlo simulations indicated that although pairs of sites within the begomovirus genome did indeed display evidence of significant linkage disequilibrium (LD; P = 0.01), the corresponding I^S^_A _was 0.0367 - a low value providing evidence that many of the polymorphic loci considered are effectively in linkage equilibrium. I^S^_A _is expected to be zero when there is no linkage among pairs of polymorphisms. The estimated I^S^_A _value for our global begomovirus dataset was, for example considerably lower than that approximated for *Helicobacter pylori *(0.0607) [31] and slightly lower than that estimated for hepatitis B virus (0.038) [32]. In both these cases the methods implemented in the program STRUCTURE has been very successfully applied and we were therefore encouraged to find that our dataset most likely displayed sufficient evidence of linkage equilibrium to enable its use in evaluating begomovirus population structure.

**Figure 1:**
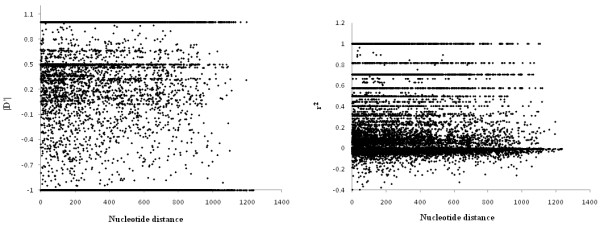
**(a b). Patterns of LD illustrated as the relationships between the distance between loci (expressed in nucleotides) and |D'| and r2, respectively**. |D'| and r2 were calculated using DnaSP (67). The existence of only weak correlations between |D'| and r2 with physical distance indicate that there is evidence of only weak LD in our begomovirus dataset.

To investigate further the degrees of LD displayed by pairs of polymorphic sites we plotted two standard measures of LD, |D'| and r^2^, against the physical distance separating pairs of sites (Fig. 1). There was no evidence of a significant decrease of LD with physical distance as indicated by the low correlation coefficients obtained for both |D'| (-0.045) and r^2 ^regressions (-0.047) against physical distance. This analysis indicated that there was no systematic LD bias in our begomovirus dataset that might seriously impact its use in the inference of gross population structure.

### Analysis of gross population structure

**Figure 2:**
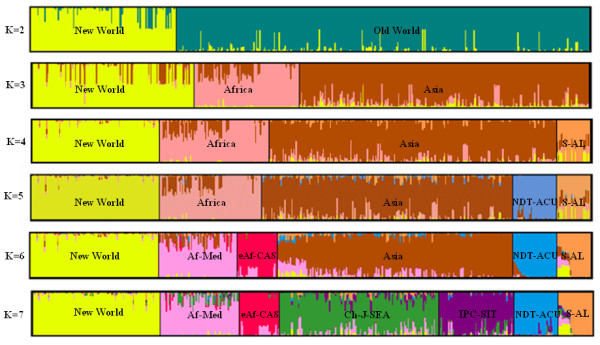
**Sequential clustering solutions obtained from K = 2 to K = 7 based on Bayesian cluster analysis of the global begomovirus dataset**. The number of clusters in a given plot is indicated by the value of K. Populations are labeled within their respective clusters. The figure shown for a given K is based on the highest likelihood run at that K. Abbreviations for the populations: New Delhi tomato-Asian cucurbit begomoviruses (NDT-ACU); Swepoviruses-Asian legumoviruses (S-AL); African-Mediterranean begomoviruses (Af-Med); East African Cassava Mosaic Viruses (eAf-CAS); Indo-Pak cotton-South Indian tomato begomoviruses (IPC-SIT).

Our initial analysis of population structure within the full begomovirus dataset aimed at discriminating between two to twelve sub-populations (i.e. K = 2 to12) failed to yield an estimate of the true optimal sub-population number in that the value of Ln P(D) increased consistently with increasing K. However, the second-order rate of change of the likelihood function (ΔK) showed a clear peak at K = 8, reflecting the existence of at least eight genetically cohesive begomovirus sub-populations each displaying distinctive nucleotide distribution patterns. Although according to ΔK, the optimal number of sub-populations for the complete begomovirus dataset was eight, we chose the more conservative K = 7 for further analysis because this number of sub-populations yielded reasonably consistent clustering in repeated analysis runs. With K = 8, either the sweet potato-infecting viruses within the larger swepovirus-Asian legumovirus sub-population (S-AL in Fig 2) or Japanese viruses within the larger China-Japan-Southeast Asia sub-population (Ch-J-SEA in Fig 2) were inconsistently consigned to sub-populations in different analysis runs.

For the sake of clarity, we named the seven sub-populations identified in the K = 7 analysis based on both the geographical location and hosts of the viruses assigned with the sub-populations. Schematic representations of the population structures revealed by our analysis are summarised in Fig 2. This figure indicates the predominant sub-populations that are discernable with sub-population numbers ranging from two to seven (i.e. K = 2 to 7). Within this figure vertical columns that contain multiple colors represent individual begomovirus sequences containing nucleotide polymorphisms that are associated with multiple different sub-populations. At K = 7, most individual sequences (393/470) were assigned to one sub-population with > 70% support for their assignment. For the remainder of this paper these seven major sub-populations will be referred to as the New World viruses, the Africa-Mediterranean viruses (Af-Med), the Swepoviruses-Asian legumoviruses (S-AL), the *East African cassava mosaic virus *group (eAf-CAS), the New Delhi tomato-Asian Cucurbit-infecting viruses (NDT-ACU), the Indo-Pak cotton-South Indian tomato viruses (IPC-SIT), and China-Japan-Southeast Asia viruses (Ch-J-SEA).

The sequential increase in population stratification noted in the analysis series with K values ranging from two through seven (Fig. 2) provides some useful insights into the relative strengths of different signals of population subdivision that are evident within the global begomovirus population. Typically, STRUCTURE will divide a dataset into its maximally divergent groups, although sample sizes and degrees of within-group diversity will also affect the exact divisions that are made [2]. In our analysis with K = 2, individuals were mostly sorted into well defined New World and Old World sub-populations. The only exceptions were the legumoviruses and swepoviruses which were not consistently classified into either group. While the New World sub-population comprised viruses from North America, Latin America, Mexico and the Caribbean, the Old World sub-population comprised Asian and Af-Med viruses. With K = 3 the Af-Med viruses were most identifiably distinct from the Asian viruses. With K = 4, the legumoviruses of Asia and the swepoviruses were together separated into a distinct sub-population (S-AL in Fig. 2). With K = 5, the eAf-CAS viruses were split from the Af-Med sub-population, to form a separate sub-population. At K = 6, tomato-infecting New Delhi viruses and Cucurbit-infecting begomoviruses together formed a new sub-population (NDT-ACU in the Fig 2). Finally, with K = 7, the Indo-Pak cotton viruses together with South Indian tomato begomoviruses (IPC-SIT in Fig 2) were separated from the China-Japan-Southeast Asian begomovirus sub-population.

Since inconsistent sub-population splits were obtained with K > 7, we attempted to identify further population structures within the seven consistently defined sub-populations obtained with K = 7. Each one of these sub-populations was treated as a main population and each was analysed separately under the admixture model with uncorrelated allele frequencies.

### Characterization of further structure within seven major sub-populations

**Figure 3:**
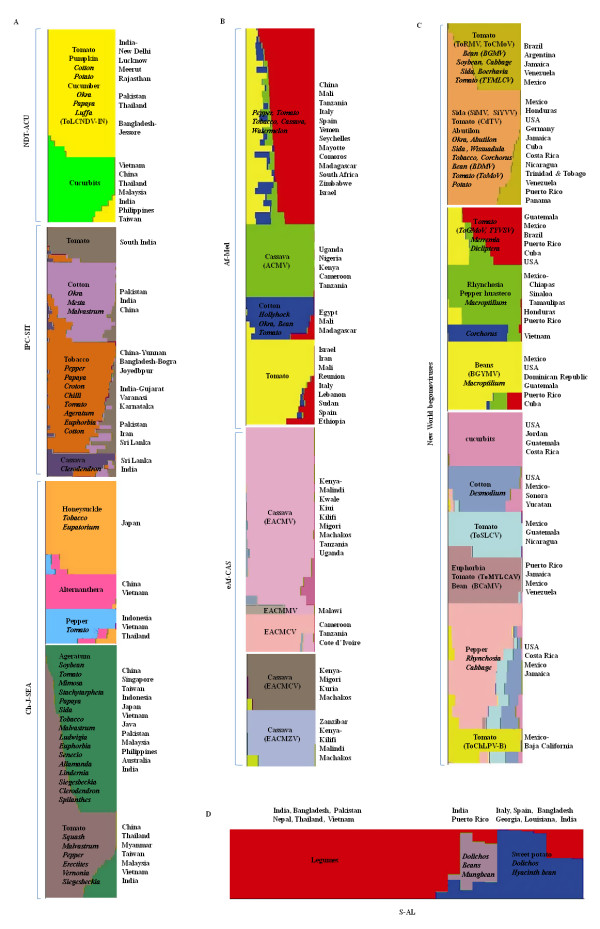
**Classification of individual viruses from major sub-populations using STRUCTURE according to membership proportions**. For convenience the sub-populations have been displayed based on their region of origin (A: Asia; B: Africa; C: New World) and Swepoviruses-Asian legumovirus group (S-AL). Different sub-populations are indicated by different colours. The hosts of the viruses are indicated within the clusters and the geographical location of member viruses is indicated along the right side of the represented population. Admixed individuals are indicated in italics. Multiple colours within individual bars are indicative of admixture. Colours that do not correspond to any minor sub-population within the major sub-populations indicate instances of inter-major sub-population admixture that could not be properly depicted within the minor sub-population plots. Correspondence of colours between different major sub-population groups is not meaningful.

A second layer of population structure analysis was performed on each of the seven major sub-populations in isolation (Fig 3). STRUCTURE analysis of four of these seven (Af-Med viruses, S-AL, NDT-ACU, and IPC-SIT viruses) yielded both consistent results in consecutive runs and some indication that an optimal number of minor (or second-level) sub-populations had been identified.

The major IPC-SIT and Af-Med sub-populations apparently each contained four genetically cohesive minor sub-populations (ΔK was maximized at K = 4; Figs 3A and 3B). Although STRUCTURE indicated that the NDT-ACU sub-population probably consists of as many as four genetically cohesive minor sub-populations (ΔK peaked at K = 4), individuals were predominantly assigned to two of these minor sub-populations with less than 50% support. In Fig 3A we present the minor sub-population structure for this group as inferred with K = 2, because with this level of subdivision almost all individuals (33/36) could be assigned to sub-populations with > 75% support Tomato-infecting viruses from New Delhi, Pakistan, and Bangladesh formed an independent cluster from cucurbit-infecting begomoviruses from all over Asia.

According to ΔK estimates, there are potentially four minor sub-populations within the major New World begomovirus population. We however chose K = 3 for further analysis because this yielded more consistent results between repeated runs. Even when the STRUCTURE analysis was performed using a linkage model we observed no improvements in clustering. For each of the three identified New World minor sub-populations, a third tier of STRUCTURE analyses was performed to identify further population structures. This third clustering hierarchy revealed a total of ten minor sub-populations within the major New World sub-population (Fig 3C). At this stage of the analyses all ten of the minor sub-populations showed consistent clustering and no further population subdivision were supported by the data.

Both of the major eAf-CAS and Ch-J-SEA sub-populations consisted of four minor sub-populations. Both of these major sub-populations showed a clear ΔK peak at K = 4 but clustering was inconsistent between runs. As membership scores were low within the identified K = 2 minor sub-populations, we performed a third tier of clustering analysis on each of these minor sub-populations separately and respectively identified four and five consistently clustered minor sub-populations within the eAf-CAS and Ch-J-SEA major sub-populations (Fig 3A and Fig 3B). Within the swepovirus-Asian legumovirus major sub-population there are apparently three minor sub-populations (Fig 3D).

### Verification of the population structure hypothesis

Collectively 34 minor sub-populations were identified within the seven major sub-populations. We tested the evidence favoring the existence of these genetically distinct minor sub-populations using AMOVA and found that all 34 were supported by a highly significant F_ST _statistic (F_ST _of 0.58; p = < 0.001). The hierarchical AMOVA of the seven major sub-populations and the 34 minor sub-populations indicated that most of the observable genetic diversity is collectively attributable to fixed genetic differences between the 34 minor sub-populations (40.96% of the diversity) and seven major sub-populations (30.65% of the diversity; Table 1).

**Table 1:** Analysis of molecular variation (AMOVA) for seven major sub-populations and 34 minor sub-populations identified within the global begomovirus meta-population.

Source of variation	Fixation indices	P-value
Among major sub-populations	(F_CT_) 0.30	< 0.001
Among minor sub-populations within major sub-populations	(F_SC_) 0.40	< 0.001
Within minor sub-populations	(F_ST_) 0.58	< 0.001

To further test whether the 34 identified minor sub-populations would be considered genetically distinct using alternative methodologies, two other statistical tests of population differentiation (Z-test of genetic differentiation implemented in DnaSP and the F_ST _permutation test implemented in ARLEQUIN) were applied to the various population partitions. The null hypothesis of no population structure was rejected with p-values < 0.001 by the Z-test [33]. Additionally, F_ST _statistics (pairwise measures of population differentiation), were calculated for each of the 34 minor sub-populations [see additional file 1]. F_ST _scores ranged from 0.09 to 0.92. Whereas an F_ST _value of 0 between two populations would indicates that they were completely undifferentiated, a score of 1 would indicate that every observable genetic difference between individual members of the two populations could be used to distinguish between the populations. Overall, a very high degree of differentiation was noted between the *Tomato chino La paz virus *group and the African cassava mosaic virus minor sub-population. (F_ST _= 0.92). The lowest degree of differentiation (F_ST _= 0.09) was observed between the New World *Tomato rugose *and *chloratic mottle *and *Tomato golden mottle virus *groups. With the exceptions highlighted (the numbers in bold) in Table S1 [see additional file 1], the various tests of genetic differentiation broadly supported the partitioning of begomovirus populations defined in our STRUCTURE analyses. Generally only comparisons between minor sub-populations with low sample sizes yielded non-significant F_ST _values_._

### Patterns of gene flow between sub-populations

The admixture model that we used in our STRUCTURE analyses assigned individuals to particular sub-populations based on their relative membership scores with respect to each of these sub-populations. These relative membership scores get encoded as colour bars in the sub-population structure maps generated by STRUCTURE (Fig 3). This representation readily allows the identification of individual sequences with polymorphic nucleotide sites that may have been derived through recombination between viruses in different sub-populations.

It is evident from the STRUCTURE plots presented in Fig 2 and Fig 3 that many individual genomes contain substantial numbers of nucleotide polymorphisms that are apparently characteristic of multiple different sub-populations. These "admixed" individuals indicate that there are probably substantial rates of gene flow between different sub-populations. Very little evidence of population admixture was observed amongst individuals assigned to the minor Asian legumovirus (red in Figure 3D), African cassava mosaic virus (green in Fig 3B), New Delhi Tomato leaf curl virus (yellow in Fig 3A) and Alternanthera yellow vein virus (pink in Fig 3A) minor sub-populations. Similarly the *Bean golden yellow mosaic virus*, *Tomato chino La paz virus *and *Pepper golden mosaic virus *minor sub-populations of the New World were found to be homogeneous with low degrees of gene flow from other sub-populations. This suggests that there is little if any recombinational integration into these sub-populations of genetic polymorphisms that are characteristic of other sub-populations.

This does not imply, however, that the members of these various minor sub-populations do not participate in recombination. There is, for example, evidence that African cassava mosaic viruses and legumoviruses have potentially contributed substantial amounts of genetic material to other minor sub-populations with which they are co-circulating.

By contrast, within the major Af-Med virus sub-population, the minor sub-population comprising begomoviruses causing diseases in African Solanaceous crops, South African cassava and Middle Eastern watermelon (indicated in red in Fig 3B) is highly admixed. There is also evidence of extensive admixture within the largest and most diverse minor sub-population within the major Ch-J-SEA sub-population (represented mostly by dark green in Fig 3A). Among the New World virus minor sub-populations there appears to have been a large degree of genetic exchange amongst the *Tomato chlorotic mottle virus-Tomato rugose mosaic virus *cluster and the *Sida mosaic virus *clusters. Similarly, the *Tomato golden mottle virus*-*Tomato yellow vein streak virus *cluster, has apparently acted as a frequent recipient of genetic material from the *Bean golden yellow mosaic virus*, *Rhyncosia golden mosaic virus *and *Pepper hausteco yellow vein virus *clusters.

## Discussion

Here we have described for the first time the fine-scale genetic structures of world-wide begomovirus DNA-A and DNA-A-like populations. We have provided clear evidence for the existence of numerous genetically cohesive begomovirus sub-populations, some of which have thus far not been appreciated as distinctive taxonomic entities. Overall, 34 largely discreet genetic entities were identified using parametric population genetic model-based clustering approaches implemented in the program STRUCTURE. The approach we have used has been very successfully applied to the study of population structure in humans [2,34,35] and many other sexually reproducing species [3,36-39]. The approach has also been prominently applied to predominantly asexual microbial species such as *Helicobacter pylori *[31], *Plasmodium falciparum *[39] and Hepatitis B virus [32]. To our knowledge, the work we have described here is the first application of this analytical approach to the study of population structure within a plant virus genus.

Consistent with current taxonomic classification of the major begomovirus lineages, our hierarchical model-based analysis of population stratification revealed that the begomoviruses can, unsurprisingly, be most broadly split into New World and Old World groups. Beyond this fundamental similarity, however, there were some potentially informative differences between the major sub-populations within these super-groups that we and others have identified. Primary among these differences is our assignment of the currently established New World swepovirus and Old World legumovirus sub-genera [20,21] to the same major sub-population within our Old World group. Second is our assignment of the currently established Meso-American and Latin American New World virus groups to the same major New-World virus sub-population, and splitting of both a major cassava infecting virus group from the established African virus group, and a NDT-ACU virus group from the established Indian group.

Despite conflicting with the current classification of swepoviruses as a distinct lineage, it is perhaps unsurprising that our analysis has indicated that swepoviruses and the legumoviruses are sister, probably Old-World, virus lineages. The swepovirus and legumovirus coat proteins are serologically closely related [40], swepoviruses have been found in both the New and Old Worlds [41-45] but have a genome organization resembling that of Old World begomoviruses [43] and there is very convincing direct evidence that swepoviruses have been donors of divergent *rep *genes found in some Old-world Africa-Mediterranean virus isolates [26]. Our analysis in fact implies that the swepoviruses are highly admixed as they possess polymorphisms that are characteristic of multiple different begomovirus sub-populations (multiple colors within individual columns of the S-AL sub-population as resolved at K = 7 in Fig 2), indicating that members of this group may also be the recombinant recipients of genetic material from viruses assigned to the major New World, Ch-J-SEA, Af-Med and eAf-CAS sub-populations. Indeed, extensive recombination in swepoviruses sampled from nature has been convincingly detected in a recent study [44].

The seven major sub-populations defined by our exploration of population genetic structure within the global begomovirus meta-population could objectively be further subdivided into 34 minor sub-populations. Importantly, our initial identification of these 34 minor sub-populations was also independently well supported by alternative non-parametric summary statistic based approaches such as AMOVA, F_ST _and Z-statistic based analyses that are also aimed at detecting and characterizing population structure.

Although geographical barriers to intercontinental movement are clearly the underlying cause of much of the observable genetic differentiation between the three main begomovirus sub-populations (K = 3 in Fig 2) it is difficult to invoke the spatial separation of populations as the only significant underlying cause of clearly structured sub-populations co-circulating in Africa (Af-Med and eAf-CAS) and Asia (S-AL, NDT-ACU, IPC-SIT and Ch-J-SEA sub-populations). Despite their close spatial association and evidence of relatively frequent recombination between members of these major sub-populations (evidenced by both the admixture observed here and patterns of recombination observed in other studies) [27,46], these Asian and African begomovirus sub-populations have still remained genetically quite distinct. This suggests that there may be some other barriers to full panmyxis (i.e. unconstrained gene flow) amongst co-circulating Asian and African begomoviruses. Amongst the most obvious candidate constraints on gene-flow amongst these sub-populations are host range and/or genetic barriers to recombination.

Accordingly, when one considers the evidence we have provided for the existence of additional population stratification within each of the seven major begomovirus sub-populations, it is apparent that in many cases viral host-ranges could be contributing to minor sub-population structure within the major sub-populations. Among the 34 genetically differentiated minor sub-populations detected, many showed strong clustering based on the hosts from which their individual members have been isolated. For example, one of the two minor sub-populations within the major NDT-ACU sub-population is entirely made up of cucurbit infecting viruses that have been sampled throughout south and Southeast Asia. Similarly, amongst the four minor sub-populations within the Ch-J-SEA major sub-population, the minor *Alternenthera*-infecting virus sub-population contains only viruses isolated from *Alternenthera spp*. Other evidence of minor sub-population stratification that may be attributable to host range restrictions on gene flow can be found in the African cotton infecting viruses, Chinese Ageratum and Tomato infecting viruses, Southern Indian and Sri Lankan tomato and cassava infecting viruses. Striking differences were also detected depending on the apparently favored host species of New World virus sub-populations. For example, the cucurbit-infecting viruses, *Bean golden yellow mosaic viruses *and *Malvaceae*-infecting viruses apparently form independent genetically isolated populations.

It must, however, be stressed firstly that very little is known about the natural host ranges of any of these virus groups and, secondly, that there exist blatant sampling biases in favor of begomovirus species/strains that cause crop diseases. The fact remains however, that whereas certain of the minor sub-populations (such as those comprising *Ageratum*-infecting viruses in the Ch-J-SEA major sub-population, Tobacco curly shoot viruses and its recombinants in the IPK SIT sub-population, ToLCNDV and its recombinants in the NDT-ACU sub-population or pepper-Mali viruses in the Af-Med sub-population) consist of viruses that have collectively been sampled from six or more different host species, others contain viruses that have only ever been sampled from one species. Interestingly, the "broad host range" minor sub-populations are also apparently more admixed than the "narrow host range" minor sub-populations. Unfortunately we cannot tell from our analysis either whether recombination has facilitated the increased host-ranges that are apparent within these sub-populations or whether increased host ranges drive increased inter-sub-population recombination frequencies.

Whereas our results are consistent with the notion that host-range differences might underlie much of the minor sub-population structure we have uncovered, it must be pointed out that viruses from many "narrow-host range" sub-populations infect the same individual plant species as viruses sampled from "broad host range" sub-populations. There are therefore presumably at least some opportunities for gene flow amongst these populations in nature. This then suggests that genetic barriers to genetic exchange, in addition to host range barriers, may underlie some of the genetic cohesiveness of many sub-populations. It is known that the viability of recombinant viruses is influenced by the relatedness of their parents and that strong purifying selection probably operates against the survival of recombinants with defective intra-protein and inter-genome region interactions [46,47]. Thus purifying selection acting against gene flow between sub-populations is likely to be at least partially responsible for the absence of admixture observed in some sub-populations. For example, despite its members co-circulating with, and infecting the same host species as other Af-Med and eAf-CAS minor sub-populations, the minor sub-population containing ACMV contains almost no evidence of admixture with any other Af-Med or eAf-CAS minor sub-populations. This result is consistent with recombination analyses which have found that whereas ACMV has occasionally donated genetic material to circulating recombinant viruses there are no known instances of predominantly ACMV genomes acting as acceptors of foreign genetic material [48]. It must, however, be stressed that while our results are consistent with the existence of genetic barriers to the flow of genetic material into sub-populations displaying low degrees of admixture, it remains to be experimentally confirmed whether or not viruses such as ACMV are particularly intolerant of inheriting genetic material from viruses belonging to different sub-populations.

Finally, we hope that our study will be perceived as complementing rather than contradicting established thinking on begomovirus taxonomy and evolution. The major and minor begomovirus sub-populations that we have identified here should provide a launch point for further population genetic studies into how population size fluctuations, selection, genetic drift, migration and gene flow have shaped currently observable patterns of begomovirus diversity. As failure to account for population structure can confound statistical tests for natural selection or population growth [49], focusing analyses on these defined sub-populations should hopefully increase the reliability and power of such tests. Whereas dissecting the relative importance of virus-vector [50,51], vector-host [52] and virus-host [12,25,53] specificities will certainly provide some valuable insights into the underlying causes of the population structures that our analysis has revealed, understanding the complex selection pressures exerted by hosts and vectors [54-56] will indicate how viruses have diversified to produce such structures. It is our intention that knowledge of these population structures should encourage more detailed studies into: (1) experimental verification of the host ranges of individuals in different sub-populations; (2) the impact of virus host ranges on gene-flow; (3) comparisons between signals of natural selection in different sub-populations and (4) dating the origins of major and minor sub-populations to track both the ancient and modern global migrations of begomoviruses.

## Materials and methods

### Sequence data

All available 690 full-length monopartite begomovirus genomes and bipartite begomovirus DNA-A genome component sequences were obtained from GeneBank using TaxBrowser. Multiple sequence alignments were constructed using ClustalW [57] and edited manually. All but one sequence within groups of sequences sharing more than 98% nucleotide identity were discarded. The resulting dataset comprised 470 complete DNA-A/DNA-A-like sequences.

### Linkage equilibrium analysis

Testing for the presence and degree of linkage disequilibrium (LD) evident in a group of sequences is a significant aspect of population genetics. Moreover, the model-based approach we used to investigate the structure of begomovirus populations assumes that different polymorphic sites along the genomes being investigated display only limited degrees of LD. From the perspective of global begomovirus diversity it is very probable that, because of the extent of inter-species genetic exchange amongst begomoviruses, many sites will be effectively in linkage equilibrium. However it was essential that we test the degree of linkage equilibrium evident within our worldwide begomovirus population sample. A null hypothesis of linkage equilibrium was tested by Monte Carlo simulations using the program LIAN (version 3.4) [58]. LIAN performs a linkage equilibrium test and yields a standardized index of association, I^S^_A_, which is a measure of the degree of haplotype-wide linkage evident in a dataset [58]. This program essentially tested the degree to which pairs of polymorphic sites within begomovirus genomes have been independently inherited (i.e. separated by recombination) during the evolutionary history of the begomoviruses as a whole. The observed variance (V_D_) of pairwise distances between groups of closely related sequences that apparently share a recent common ancestry (these are called haplotypes), is computed and compared to the variance expected when all loci are in linkage equilibrium (V_E_). Only polymorphic sites were included for the analysis and a 5% critical value was obtained as described [59]. In addition, traditional measures of LD namely |D'| [60] and r^2 ^[61], were estimated using DnaSP [62].

### Population structure analysis

Global begomovirus population structure was investigated using the program STRUCTURE (Version 2.0) [1]. This program applies a Bayesian model-based approach to analyse population structure and identifies both groups of genetically similar individuals and divergent populations of individuals on the basis of allele frequencies.

In the beginning, *ad hoc *STRUCTURE runs were performed to determine the optimum number of iterations for the initial burn-in and estimation phases of the analysis so as to ensure the reliability of posterior probability estimates. Burn-in and parameter estimation iterations ranging from 20,000 to 40,000 did not yield significantly different results. From these preliminary analyses we determined that an initial burn-in of 40,000 iterations followed by 40,000 iterations for parameter estimation was sufficient. To estimate the number of populations (the K parameter), the begomovirus dataset was analyzed allowing the value of K to vary from 1 to 12. Five independent runs were carried out for each K value (equating to 60 runs in total). As advised in the STRUCTURE user's manual, we set most of the parameters to their default values [63]. Specifically, we chose the admixture model with the option of correlated allele frequencies between populations [31]. This model can account both for some individuals having mixed ancestry and for allele frequencies in sub-populations being similar due to admixture or shared ancestry. This model is an appropriate choice in that there is ample evidence available for both rampant begomovirus recombination and substantial movement of begomoviruses across different regions of the world. Indeed, this model is also considered best in cases where population structure is subtle [31]. We co-estimated the degree of admixture (the alpha parameter) from the data. When alpha is close to zero, most individuals fall into clearly defined sub-populations but when alpha > 1 most individuals carry a range of alleles that make it difficult to unambiguously assign them to particular sub-populations [31]. The lambda parameter describing the distribution of allele frequencies was set to one. The optimum number of sub-populations (K_opt_) was identified as previously described [64].

For K_opt_, each individual was then assigned to one of the sub-populations, according to their respective estimated membership scores (ranging from 0 to 1 for each individual sequence for each sub-population and summing to 1 for each individual across all sub-populations) for each of the different sub-populations. Individuals that could be assigned to two or more different sub-populations each with membership scores of 0.15 or higher were considered to be admixed. It is important to note that despite our expecting the admixture model to identify the correct number of sub-populations we also expected it to generally overestimate the proportion of admixed individuals by ignoring linkage between polymorphic nucleotide sites that were physically very close to one another within the begomovirus genomes. We also applied the linkage model for K_opt _in order to account for potential physical linkage between loci when refining the sub-population assignment of difficult to assign individuals. For this model the burn-in and MCMC run lengths were set at 20000 and 40000 respectively, with a 10000 iteration admixture burn-in length.

### Sublevel clustering

We used the first hierarchical sub-population cluster classification inferred by STRUCTURE to study finer-scale clustering within major begomovirus sub-populations. Each of the seven established major begomovirus sub-populations was considered as a major sub- population and analysed separately under the admixture model with uncorrelated allele frequencies (and the value of λ inferred for each sub-population). We used ΔK, an *ad hoc *parameter as described in [64] to determine the optimum (or at least the most probable) number of sub-populations. The number of populations was fixed at a lower K wherever firstly, the assignment of particular sequences to sub-populations was inconsistent over different runs and, secondly, whenever no individual sequences at the highest ΔK exhibited membership probability scores > 70%.

### Molecular variation, population differentiation and Genetic divergence

The population stratifications inferred by STRUCTURE were tested by analysis of molecular variance (AMOVA) as implemented in ARLEQUIN (ver. 3.0) [65]. AMOVA measures the partitioning of variance at different levels of population subdivision, and yields F-statistics known as fixation indices (or F_ST _statistics). The fixation indices estimated from the begomovirus sequence analyses were tested using a non-parametric permutation approach as described in [66]. Furthermore the significance of F_ST _based estimates of population structure was also tested in ARLEQUIN using a permutation test (with 1000 randomised iterations) as in [67]. Also, DnaSP (version 4.0) [62] was used to estimate Z test statistics of genetic differentiation [33]. Permutation tests with 10 000 replicates were performed to test the significance of these statistics.

## Competing interests

The authors declare that they have no competing interests.

## Authors' contributions

HCP conceived, designed the study. HCP, DPS, AV, MS, BS and MR performed sequence alignments and population structure analysis. HCP and DPM interpreted data and wrote the manuscript. All authors have read and approved the final manuscript.

## Supplementary Material

Additional file 1 **Table S1. Differentiation between the 34 minor begomovirus sub-populations identified in this study**. Data provided represent pairwise measures of population differentiation (F_ST_). Non significant F_ST _values based on permutation test are highlighted. Click here for file
