# Supplementary material for: The population genomics of begomoviruses: global scale population structure and gene flow
Source: Virol J. 2010 Sep 10;7:220. doi: 10.1186/1743-422X-7-220 (PMC2945956; doi:10.1186/1743-422X-7-220)
Supplement: Additional file 1 — Table S1. Differentiation between the 34 minor begomovirus sub-populations identified in this study. Data provided represent pairwise measures of population differentiation (FST). Non significant FST values based on permutation test are highlighted. [file 1743-422X-7-220-S1.DOC]

Table S1 Differentiation between the 34 minor begomovirus sub-populations identified in this study (Non significant F_ST_ values based on permutation test are highlighted).

| Major sub-population | Minor sub-population (number of sequences) | 1 | 2 | 3 | 4 | 5 | 6 | 7 | 8 | 9 | 10 | 11 | 12 | 13 | 14 | 15 | 16 | 17 | 18 | 19 | 20 | 21 | 22 | 23 | 24 | 25 | 26 | 27 | 28 | 29 | 30 | 31 | 32 | 33 |
| --- | --- | --- | --- | --- | --- | --- | --- | --- | --- | --- | --- | --- | --- | --- | --- | --- | --- | --- | --- | --- | --- | --- | --- | --- | --- | --- | --- | --- | --- | --- | --- | --- | --- | --- |
| Africa-Middle East Asia | 1. Pepper- Mali group(33) | 0.00 |  |  |  |  |  |  |  |  |  |  |  |  |  |  |  |  |  |  |  |  |  |  |  |  |  |  |  |  |  |  |  |  |
|  | 2. African Cassava(12) | 0.43 | 0.00 |  |  |  |  |  |  |  |  |  |  |  |  |  |  |  |  |  |  |  |  |  |  |  |  |  |  |  |  |  |  |  |
|  | 3. *Malvaceaeceous* (7) | 0.31 | 0.79 | 0.00 |  |  |  |  |  |  |  |  |  |  |  |  |  |  |  |  |  |  |  |  |  |  |  |  |  |  |  |  |  |  |
|  | 4. Tomato -Middle East(14) | 0.22 | 0.71 | 0.57 | 0.00 |  |  |  |  |  |  |  |  |  |  |  |  |  |  |  |  |  |  |  |  |  |  |  |  |  |  |  |  |  |
|  |  |  |  |  |  |  |  |  |  |  |  |  |  |  |  |  |  |  |  |  |  |  |  |  |  |  |  |  |  |  |  |  |  |  |
| New Delhi Tomato-Asian Cucurbits | 5. Tomato-New Delhi(25) | 0.54 | 0.85 | 0.77 | 0.74 | 0.00 |  |  |  |  |  |  |  |  |  |  |  |  |  |  |  |  |  |  |  |  |  |  |  |  |  |  |  |  |
|  | 6. Cucurbits-Southeast Asia(12) | 0.47 | 0.82 | 0.70 | 0.67 | 0.47 | 0.00 |  |  |  |  |  |  |  |  |  |  |  |  |  |  |  |  |  |  |  |  |  |  |  |  |  |  |  |
|  |  |  |  |  |  |  |  |  |  |  |  |  |  |  |  |  |  |  |  |  |  |  |  |  |  |  |  |  |  |  |  |  |  |  |
| Swepovirus- Legumovirus | 7. Mungbean –Asia(19) | 0.55 | 0.80 | 0.70 | 0.70 | 0.77 | 0.72 | 0.00 |  |  |  |  |  |  |  |  |  |  |  |  |  |  |  |  |  |  |  |  |  |  |  |  |  |  |
|  | 8. Dolichos group(3) | 0.18 | **0.72** | **0.42** | 0.43 | 0.57 | **0.34** | 0.59 | 0.00 |  |  |  |  |  |  |  |  |  |  |  |  |  |  |  |  |  |  |  |  |  |  |  |  |  |
|  | 9. Swepovirus(7) | 0.40 | 0.67 | 0.49 | 0.54 | 0.68 | 0.56 | 0.54 | **0.24** | 0.00 |  |  |  |  |  |  |  |  |  |  |  |  |  |  |  |  |  |  |  |  |  |  |  |  |
|  |  |  |  |  |  |  |  |  |  |  |  |  |  |  |  |  |  |  |  |  |  |  |  |  |  |  |  |  |  |  |  |  |  |  |
| New World | 10. Tomato Rugose & chlorotic mottle(19) | 0.47 | 0.68 | 0.57 | 0.59 | 0.68 | 0.60 | 0.61 | 0.42 | 0.45 | 0.00 |  |  |  |  |  |  |  |  |  |  |  |  |  |  |  |  |  |  |  |  |  |  |  |
|  | 11. Sida virus group(32) | 0.53 | 0.72 | 0.64 | 0.64 | 0.71 | 0.66 | 0.67 | 0.53 | 0.55 | 0.22 | 0.00 |  |  |  |  |  |  |  |  |  |  |  |  |  |  |  |  |  |  |  |  |  |  |
|  |  |  |  |  |  |  |  |  |  |  |  |  |  |  |  |  |  |  |  |  |  |  |  |  |  |  |  |  |  |  |  |  |  |  |
|  | 12. Tomato golden mottle group(7) | 0.47 | 0.76 | 0.60 | 0.62 | 0.75 | 0.66 | 0.65 | **0.39** | 0.42 | **0.09** | 0.27 | 0.00 |  |  |  |  |  |  |  |  |  |  |  |  |  |  |  |  |  |  |  |  |  |
|  | 13. Pepper & Rhynchosia group(7) | 0.51 | 0.82 | 0.68 | 0.69 | 0.79 | 0.72 | 0.70 | **0.50** | 0.49 | 0.33 | 0.42 | **0.34** | 0.00 |  |  |  |  |  |  |  |  |  |  |  |  |  |  |  |  |  |  |  |  |
|  | 14. Corchorus-Vietnam(2) | 0.51 | **0.89** | **0.70** | **0.71** | **0.84** | **0.76** | **0.72** | **0.38** | **0.39** | **0.41** | 0.52 | **0.39** | **0.47** | **0.00** |  |  |  |  |  |  |  |  |  |  |  |  |  |  |  |  |  |  |  |
|  | 15. Bean golden yellow mosaic virus(8) | 0.56 | 0.87 | 0.75 | 0.74 | 0.83 | 0.77 | 0.74 | **0.62** | 0.57 | 0.34 | 0.42 | 0.35 | 0.55 | **0.68** | 0.00 |  |  |  |  |  |  |  |  |  |  |  |  |  |  |  |  |  |  |
|  |  |  |  |  |  |  |  |  |  |  |  |  |  |  |  |  |  |  |  |  |  |  |  |  |  |  |  |  |  |  |  |  |  |  |
|  | 16. Cucurbits(6) | 0.55 | 0.86 | 0.72 | 0.73 | 0.82 | 0.76 | 0.74 | **0.56** | 0.53 | 0.35 | 0.48 | **0.39** | 0.56 | **0.60** | 0.59 | 0.00 |  |  |  |  |  |  |  |  |  |  |  |  |  |  |  |  |  |
|  | 17. Cotton leaf crumple group(3) | 0.53 | 0.89 | **0.73** | 0.73 | 0.84 | **0.77** | 0.74 | **0.48** | **0.47** | 0.31 | 0.38 | **0.34** | **0.54** | **0.57** | **0.62** | **0.43** | 0.00 |  |  |  |  |  |  |  |  |  |  |  |  |  |  |  |  |
|  | 18. ToSLCV-GT group(5) | 0.56 | 0.89 | 0.75 | 0.74 | 0.84 | 0.78 | 0.75 | **0.58** | 0.54 | 0.35 | 0.46 | 0.39 | **0.58** | **0.66** | 0.64 | **0.39** | **0.46** | 0.00 |  |  |  |  |  |  |  |  |  |  |  |  |  |  |  |
|  | 19. Euphorbia(5) | 0.55 | 0.88 | 0.74 | 0.74 | 0.83 | 0.77 | 0.74 | **0.56** | 0.53 | 0.33 | 0.46 | **0.37** | 0.57 | **0.63** | 0.61 | **0.25** | **0.43** | **0.33** | 0.00 |  |  |  |  |  |  |  |  |  |  |  |  |  |  |
|  | 20. Pepper (9) | 0.57 | 0.85 | 0.73 | 0.73 | 0.82 | 0.76 | 0.73 | **0.60** | 0.57 | 0.36 | 0.48 | 0.41 | 0.57 | **0.64** | 0.59 | 0.38 | **0.45** | 0.44 | **0.37** | 0.00 |  |  |  |  |  |  |  |  |  |  |  |  |  |
|  | 21. Tomato china La Paz(4) | 0.55 | 0.92 | **0.77** | 0.75 | 0.85 | 0.80 | 0.76 | **0.59** | **0.53** | 0.31 | 0.47 | **0.36** | **0.61** | **0.73** | 0.68 | 0.65 | **0.70** | **0.64** | **0.67** | 0.64 | 0.00 |  |  |  |  |  |  |  |  |  |  |  |  |
|  |  |  |  |  |  |  |  |  |  |  |  |  |  |  |  |  |  |  |  |  |  |  |  |  |  |  |  |  |  |  |  |  |  |  |
| Indo-Pak cotton-south Indian tomato virus group | 22. Tomato-south India(9) | 0.43 | 0.81 | 0.67 | 0.63 | 0.72 | 0.63 | 0.70 | **0.43** | 0.53 | 0.57 | 0.64 | 0.62 | 0.69 | **0.73** | 0.75 | 0.73 | **0.74** | 0.76 | 0.75 | 0.74 | **0.78** | 0.00 |  |  |  |  |  |  |  |  |  |  |  |
|  | 23. Indo-Pak cotton(20) | 0.39 | 0.69 | 0.56 | 0.56 | 0.62 | 0.55 | 0.66 | 0.36 | 0.51 | 0.55 | 0.61 | 0.57 | 0.63 | **0.64** | 0.67 | 0.67 | 0.66 | 0.68 | 0.67 | 0.67 | 0.68 | 0.47 | 0.00 |  |  |  |  |  |  |  |  |  |  |
|  | 24. Tobacco curly shoot virus group(28) | 0.37 | 0.64 | 0.54 | 0.52 | 0.56 | 0.49 | 0.63 | 0.31 | 0.50 | 0.54 | 0.60 | 0.57 | 0.61 | **0.62** | 0.65 | 0.64 | 0.63 | 0.65 | 0.65 | 0.66 | 0.66 | 0.32 | 0.35 | 0.00 |  |  |  |  |  |  |  |  |  |
|  | 25. Cassava Indo-Lanka(6) | 0.37 | 0.76 | 0.59 | 0.59 | 0.67 | 0.56 | 0.67 | **0.27** | 0.43 | 0.52 | 0.60 | 0.53 | 0.62 | **0.60** | 0.70 | 0.66 | **0.65** | **0.69** | 0.68 | 0.68 | **0.70** | 0.53 | 0.43 | 0.39 | 0.00 |  |  |  |  |  |  |  |  |
|  |  |  |  |  |  |  |  |  |  |  |  |  |  |  |  |  |  |  |  |  |  |  |  |  |  |  |  |  |  |  |  |  |  |  |
| *East African Cassava virus* group | 26. EACMV Cameroon(4) | 0.38 | 0.88 | 0.70 | 0.64 | 0.82 | 0.76 | 0.74 | **0.50** | **0.51** | 0.59 | 0.67 | **0.64** | **0.73** | **0.80** | **0.81** | **0.77** | **0.80** | **0.82** | **0.80** | 0.78 | **0.87** | 0.74 | 0.63 | 0.59 | **0.66** | 0.00 |  |  |  |  |  |  |  |
|  | 27. EACMV-Kenya(20) | 0.43 | 0.85 | 0.77 | 0.70 | 0.83 | 0.81 | 0.79 | 0.71 | 0.70 | 0.69 | 0.73 | 0.77 | 0.82 | **0.87** | 0.86 | 0.85 | 0.87 | 0.87 | 0.86 | 0.84 | 0.88 | 0.80 | 0.70 | 0.66 | 0.77 | 0.56 | 0.00 |  |  |  |  |  |  |
|  | 28. EACMKV(5) | 0.32 | 0.89 | **0.72** | 0.64 | 0.83 | 0.78 | 0.76 | **0.56** | **0.56** | 0.61 | 0.68 | **0.68** | 0.77 | **0.86** | 0.84 | **0.81** | **0.86** | **0.86** | **0.84** | 0.80 | **0.91** | **0.77** | 0.63 | 0.59 | **0.70** | **0.79** | 0.64 | 0.00 |  |  |  |  |  |
|  | 29. EACMKV-(5) | 0.33 | 0.89 | 0.72 | 0.64 | 0.83 | 0.78 | 0.76 | **0.56** | **0.55** | 0.61 | 0.68 | **0.67** | **0.76** | **0.86** | 0.84 | **0.81** | **0.86** | **0.86** | **0.84** | 0.80 | **0.90** | 0.76 | 0.63 | 0.59 | 0.69 | **0.80** | 0.66 | **0.73** | 0.00 |  |  |  |  |
|  |  |  |  |  |  |  |  |  |  |  |  |  |  |  |  |  |  |  |  |  |  |  |  |  |  |  |  |  |  |  |  |  |  |  |
| China-Japan-Southeast Asia virus group | 30. Japan virus group(19) | 0.42 | 0.73 | 0.61 | 0.59 | 0.72 | 0.65 | 0.68 | **0.25** | 0.54 | 0.59 | 0.64 | 0.62 | 0.67 | **0.69** | 0.72 | 0.71 | 0.70 | 0.72 | 0.71 | 0.71 | 0.73 | 0.62 | 0.56 | 0.52 | 0.57 | 0.67 | 0.73 | 0.67 | 0.68 | 0.00 |  |  |  |
|  | 31. Pepper-Indonesia(7) | 0.36 | 0.73 | 0.54 | 0.55 | 0.70 | 0.60 | 0.63 | **0.27** | 0.39 | 0.50 | 0.59 | 0.50 | 0.58 | **0.55** | 0.66 | 0.62 | **0.60** | 0.64 | **0.63** | 0.64 | **0.65** | 0.55 | 0.49 | 0.47 | 0.47 | **0.60** | 0.74 | 0.64 | 0.64 | 0.54 | 0.00 |  |  |
|  | 32*. Alternanthera* group(7) | 0.49 | 0.89 | 0.75 | 0.72 | 0.83 | 0.78 | 0.75 | **0.60** | 0.58 | 0.62 | 0.69 | **0.69** | 0.76 | **0.83** | 0.83 | 0.81 | **0.84** | 0.84 | 0.83 | 0.80 | 0.88 | 0.76 | 0.64 | 0.60 | 0.70 | 0.85 | 0.86 | **0.88** | 0.88 | 0.66 | 0.63 | 0.00 |  |
|  | 33. Tomato-China(32) | 0.34 | 0.62 | 0.51 | 0.48 | 0.60 | 0.53 | 0.61 | 0.26 | 0.48 | 0.54 | 0.59 | 0.55 | 0.60 | **0.61** | 0.64 | 0.63 | 0.62 | 0.64 | 0.63 | 0.64 | 0.64 | 0.48 | 0.45 | 0.37 | 0.47 | 0.56 | 0.63 | 0.57 | 0.57 | 0.40 | 0.45 | 0.55 | 0.00 |
|  | 34. Ageratum group(69) | 0.23 | 0.47 | 0.37 | 0.35 | 0.47 | 0.41 | 0.50 | **0.11** | 0.39 | 0.45 | 0.50 | 0.45 | 0.50 | 0.49 | 0.53 | 0.54 | 0.52 | 0.54 | 0.53 | 0.54 | 0.53 | 0.37 | 0.34 | 0.30 | 0.34 | 0.43 | 0.48 | 0.42 | 0.42 | 0.31 | 0.31 | 0.41 | 0.18 |
